# The Effect of Global Warming on Complex Disorders (Mental Disorders, Primary Hypertension, and Type 2 Diabetes)

**DOI:** 10.3390/ijerph19159398

**Published:** 2022-07-31

**Authors:** Sarya Natur, Odeya Damri, Galila Agam

**Affiliations:** Department of Clinical Biochemistry and Pharmacology and Psychiatry Research Unit, Faculty of Health Sciences and the Zlotowski Center for Neuroscience, Ben-Gurion University of the Negev, Beer-Sheva 8410501, Israel; sarya@post.bgu.ac.il (S.N.); odeyad@post.bgu.ac.il (O.D.)

**Keywords:** global warming, climate change, mental disorders, primary hypertension, type 2 diabetes

## Abstract

Multiple studies imply a strong relationship between global warming (GW) and complex disorders. This review summarizes such reports concentrating on three disorders—mental disorders (MD), primary hypertension, and type 2 diabetes (T2D). We also attempt to point at potential mechanisms mediating the effect of GW on these disorders. Concerning mental disorders, immediate candidates are brain levels of heat-shock proteins (HSPs). In addition, given that heat stress increases reactive oxygen species (ROS) levels which may lead to blood–brain barrier (BBB) breakdown and, hence, enhanced protein extravasation in the brain, this might finally cause, or exacerbate mental health. As for hypertension, since its causes are incompletely understood, the mechanism(s) by which heat exposure affects blood pressure (BP) is an open question. Since the kidneys participate in regulating blood volume and BP they are considered as a site of heat-associated disease, hence, we discuss hyperosmolarity as a potential mediator. In addition, we relate to autoimmunity, inflammation, sodium excretion, and HSP70 as risk factors that might play a role in the effect of heat on hypertension. In the case of T2D, we raise two potential mediators of the effect of exposure to ambient hot environment on the disease’s incidence—brown adipose tissue metabolism and HSPs.

## 1. Introduction

Global warming (GW) is one of the components of climate change driven by human emission of greenhouse gases, which eventually results in massive shifts in weather patterns [1,2]. The globe has experienced prior periods of climatic change; however, since the mid-20th century humans have had unprecedented impact on earth’s climate system and caused change on a global scale [2].

Complex disorders are caused by a combination of genetic, environmental, and lifestyle factors, most of which have not yet been identified. Most diseases fall into this category, including several congenital defects and several adult-onset diseases [3].

Mental disorders (MDs) are behavioral or mental impairment resulting in aberrant personal functioning [4]. These psychological syndromes are characterized by significant disturbance in an individual’s cognition, emotion regulation, or behavior, mirroring psychological, biological, and developmental dysfunction. Two central categories of mental disorders to which we relate are: (1) Affective (emotion/mood) disorders that include sustained intense sadness, melancholia, up to despair (unipolar depression), and bipolar disorder (manic depression) involving alternating abnormally “high”, normal and depressed mood states; (2) anxiety types such as phobias, generalized anxiety, social anxiety, and post-traumatic stress disorder. In the United States (US), one in five adults experiences mental illness each year [5]. MD patients suffer from support needs, institutionalization, discrimination, social exclusion, and adverse effects of medications.

Primary hypertension is a common condition resulting from a high and long-term force of the blood against one’s artery walls, causing health problems. The term primary hypertension (or essential or idiopathic hypertension) relates to the form which lacks identifiable secondary cause. It affects 85% of those with high blood pressure; the remaining 15% include those of various causes of secondary hypertension [6]. Primary hypertension tends to be familial and, similarly to other complex disorders, is believed to be the consequence of an interaction between environmental and genetic factors. The prevalence of primary hypertension increases with age.

Type 2 diabetes (T2D) (formerly adult-onset diabetes), a typically chronic illness, is characterized by high blood sugar, insulin resistance, and relative lack of insulin [7]. Due to complications such as a significantly higher risk of cardiovascular disease, ischemic heart disease and stroke, an increased rate of leg amputations, and increased hospitalizations rate, it results in a ten-year shorter life expectancy [8,9].

Multiple studies imply a strong relationship between GW and complex disorders. This scoping review summarizes such reports and attempts to point at potential biochemical and ultrastructural mechanisms mediating the effect of GW on these disorders.

## 2. Global Warming and Mental Disorders

Investigators from MIT, Harvard, and other top American research institutions reported a largescale study in which they retrospectively empirically coupled meteorological and climate data with reported mental health (MH) difficulties of nearly two million randomly sampled US residents between 2002 and 2012 [10]. They found that raising temperatures from between 25 °C and 30 °C to >30 °C significantly increased the prevalence of MH issues, providing quantitative support to the notion that GW poses threats to human MH.

A Chinese study [11] evaluated the short-term effects of daily mean temperature on hospital admissions due to MDs. Regarding 18.3 °C as the median temperature, the authors found a significant positive association between the temperature above threshold (24.6 °C) and emergency department visits and hospital admissions due to MD. The relative risk of extreme hot temperatures (33.1 °C, percentile 99%) compared to the median temperature was 1.27. Other studies found that cases with mania increased significantly with high temperatures, while the number of cases of depression increased in the winter [12,13,14]. Interestingly, women were more vulnerable than men, with no difference between the groups’ age [12]. In contrast, in another study that evaluated the association between long-term exposure to elevated temperature and major depressive disorder (MDD) incidence [15] it was found that elderly subjects were more vulnerable to heat exposure, and that females were less susceptible than males to heat-related MDD (Hazard Ratio = 1.14 *vs*. 1.18).

Extreme weather and other climate-related events affect communities’ and individuals’ psychological well-being, including acute traumatic stress, criminal behavior [16], chronic MDs such as depression, post-traumatic stress disorder (PTSD), sleep difficulties, social avoidance, irritability, and drug or alcohol abuse [17,18,19]. Climate change may affect MH both by direct and by indirect pathways [18,20], resulting in serious problems, even increased suicide mortality [21].

It may be summarized that exposure to extreme hot temperature is considered a significant risk factor for MDs, but the pathophysiological processes linking between the two are yet unraveled. In the following section we raise potential mechanisms linking exposure to heat and MH morbidity.

## 3. Global Warming and Mental Disorders—Potential Mechanisms

### 3.1. The Heat Shock Proteins (HSPs) System

It is well-known that vertebrates respond to chronic exposure to hot ambient temperature by operating a phenotypic adaptation and that plasticity of the thermoregulatory center in the hypothalamus is vital for heat acclimation [22,23]. To execute adaptation to heat, molecular and cellular mechanisms (see below), shifts in temperature thresholds of heat dissipation and thermal injury are required, associated with enhanced inducible cytoprotective networks. These include changes in the expression of HSP70, heat shock factor (HSF)1, hypoxia-inducible factor (HIF)1α, and posttranslational histone modifications in the promoters of HSP70 and HSP90 in a variety of tissues [22]. Of particular interest is HSP70. There are a variety of human HSP70 isoforms encoded by a multi-gene family and there are multiple HSP proteins with molecular masses of 72, 73, 75, and 78-kDa [24,25]. HSP72 is the most heat-sensitive and highly inducible [26]. Under unstressed conditions, monomeric HSF-1 is attached to HSP72 in the cytoplasm while under stress conditions, e.g., heat shock, the following cascade of events may take place: misfolded thermally denatured proteins accumulate in the cytoplasm. This induces dissociation of the complex of HSF-1 and HSP72, then free HSP72 binds the denatured proteins facilitating refolding, thus restoring cellular homeostasis since the cytoskeletal structure is maintained, guaranteeing the cell’s survival and function [26]. Consequently, HSF-1 monomer trimerizes, undergoes phosphorylation, migrates to the nucleus, and attaches to the heat shock element (HSE). The latter is located in the promoter region of HSP genes [27]. This results in increased transcription and expression of heat shock genes’ mRNA [28]. Protein translation then increases intracellular HSP72 levels, an increase leading to thermotolerance [29].

### 3.2. The Link between Heat Stress (HS) and Oxidative Stress

The brain embodies ~2% of the mass of the human body. However, in the resting state, it utilizes about 20% of the total oxygen consumed by the organism since neurons require several orders of magnitude more energy than other cells [30]. Most of the energy used for neuronal metabolism is required to restore membrane potential following electrical discharge [31], suggesting a relationship between metabolic and electrical neural activity, macromolecules synthesis and protons transport across mitochondrial membranes. On top of the neurons, glial and endothelial cells, numbers of which greatly exceed those of brain neuronal cells, are also metabolically active [32]. Given that the energy used for brain metabolism is converted into heat, heat production is an essential characteristic of the metabolic activity of the brain. Reactive oxygen species (ROS), by-products of oxygen metabolism during respiration, are produced continuously in aerobic organisms [33]. An imbalance between ROS production and antioxidant defense results in oxidative stress. HS is an environmental factor provoking mitochondrial ROS production [34]. Other causes for elevated ROS levels following hyperthermia include decreased superoxide dismutase 1 (SOD-1) activity [35] and transition metal ions overproduction. The latter form superoxide anions by transferring an electron to oxygen [36]. Increased ROS levels due to HS [34] might also lead to mitochondrial dysfunction, altered metal homeostasis and reduced antioxidant defense, all of which may exacerbate MD [37].

### 3.3. Heat Stress and the Blood-Brain Barrier (BBB)

In HS disorder, the thermal information processing system (IPS) which is regulated in the hypothalamus is aberrant [21,38]. Excessive heat overload harms the cooling mechanisms resulting in clinical hyperthermia. Consequently, depending on its magnitude and severity, it results in brain dysfunction. Neurochemicals and pathways of cell signaling affect the IPS and cell injury, apparently by altering BBB function [35,39]. A crucial event which determines the extent of cell injury/cell death/brain pathology is serum proteins leakage across the BBB, causing vasogenic brain edema [40]. Hanin has suggested that the drastic increase in the frequency of neurological symptoms which occurred in Israeli soldiers following pyridostigmine ingested as an antidote of nerve gas poisoning during the Gulf War, was due to the development of leaky barriers as a result of their exposure to conditions of hot weather existing in that region [40]. This result corroborated earlier findings that mild hyperthermia causes BBB breakdown enhancing brain protein extravasation [41]. A protein playing a role in sensing the ambient temperature is TRPV4 (thermosensitive transient receptor potential vanilloid 4) which belongs to the superfamily of the transient receptor potential (TRP) ion channels. It is a Ca^2+^-permeable nonselective cation channel expressed in the brain [42] activated at body temperature around 25–34 °C [43,44]. In the central nervous system (CNS), TRPV4 is expressed in cells including neurons [45], glial cells [46,47], and vascular endothelial cells [48] and plays a pivotal role in CNS disorders [49]. Importantly, it was reported to modulate the BBB [49,50]. Disrupted BBB has been linked to a variety of MDs [41].

To sum up, although there is no clear-cut understanding what processes mediate the effects of exposure to heat on MH, the first possibility that comes to mind is brain levels of HSPs. In addition, given that HS increases ROS levels which may lead to BBB breakdown and, hence, enhance protein extravasation in the brain, this might finally cause, or exacerbate MH.

## 4. Global Warming and Primary Hypertension

Studies from Germany, China, and other sources indicate that ambient temperature is associated with blood pressure (BP) [51,52,53]. In general, high ambient temperatures decrease BP in young and middle-aged adults [54,55], lower temperatures seem to increase adults’ BP, and HS increases human morbidity and mortality as compared with normothermic conditions [56,57]. Thus, in the China Hypertension Survey study carried out from October 2012 to December 2015 [58], which included 451,770 individuals, the surveyed seasons were divided into warm and cold ones (subtropical, temperate monsoon and temperate continental zones). Overall, a 10 °C decrease in ambient temperature was statistically associated with 0.74 mmHg and 0.60 mmHg rise in systolic- and diastolic-BP (SBP and DBP), respectively [58]. Another study, consisting of men aged 53–100 years, estimated the effect of three different temperature-related variables—ambient, apparent, and dew point temperature on repeated measures of DBP and SBP every 3–5 years [51]. The results indicated a relationship between DBP and ambient and apparent temperature [59]. The authors suggested that *an increase in BP could be a mechanism behind cold-related, but not heat-related, cardiovascular mortality* [59]. It seems that during periods of cold weather, an increase in BP variability may complicate the diagnosis and management of hypertension, thereby contributing to the high cardiovascular mortality observed in winter [60,61]. Cardiovascular mortality, frequently associated with high BP, has been linked to changes in outdoor temperature [51,62]. Intriguingly, indoor temperature appeared to have a stronger effect on BP than outdoor temperature [61]. The causes of hypertension are incompletely understood and, hence, the mechanism(s) by which heat exposure affects BP is an open question. Below, we summarize some risk factors for the disease that might play a role in the effect of heat on hypertension.

## 5. Global Warming and Primary Hypertension—Potential Mechanisms

It is well established that kidneys’ function is closely related to BP [57,63].

### 5.1. Hyperosmolarity

Exposure to high temperatures causes dehydration and blood hyperosmolality [63,64]. The kidneys participate in regulating blood volume and BP and are therefore, considered as a site of heat-associated disease [63,65]. Additionally, it has been reported that climate change elicits variations in biochemical parameters of kidney function [65].

### 5.2. Autoimmunity, Inflammation, Sodium Excretion and HSP70

It becomes apparent that autoimmunity plays a central role in essential hypertension [55]. Infiltration of immune cells in the kidney is a robust finding, associated with aberrant sodium excretion [51]. Approximately one third of the essential hypertensive population is responsive to sodium intake [66]. Furthermore, impairment of pressure-natriuresis relationship induced by inflammation of vascular relaxation and of the sympathetic nervous system are all involved in high BP induction [67]. In this respect, there is a strong relationship between inflammation, in general, T-cells, in particular, along with autoimmune reactivity on the one hand, and HSPs on the other in BP (dys)regulation [68]. As for the T cells, a crucial factor is the balance between T-cell-induced inflammation and T-cell suppressor responses [69]. Thus, autoimmunity in the kidney and arteries could stem from inflammation due to neoantigens expression, and from translocation of intracellular immunogenic proteins (e.g., HSPs), supporting the concept that HSPs are immunodominant molecules and that autoimmune reactivity against HSP70 might be a contributing factor playing a role in the pathophysiology of hypertension [55]. In addition, it has been shown that overexpression of HSP70 in the kidney in salt-driven hypertension induces T cells to present a CD4 clonal response [51]. Given the role of HSPs, and HSP70 in particular, in the response to exposure to hot ambient temperature [55], it is conceivable that these proteins mediate the effect of heat on hypertension.

To sum-up, inter-relationship between autoimmunity and sodium excretion at the kidney level, and autoimmune reactivity directed specifically against HSP70, as well as inflammation-induced dysfunctional vascular relaxation are all potential players as mediating the effect of heat on hypertension.

## 6. Global Warming and Type 2 Diabetes

Here we summarize three recent epidemiological studies from different continents. Overall, the studies imply an association between the ambient temperature and the risk for diabetes, reflected by various measures. In one of the studies [70], data of daily diabetes mortality of four tropical cities (in the Philippines) was collected between 2006 and 2011 in parallel with meteorological data from the National Oceanic and Atmospheric Administration. The study found that high diabetes risk occurs by both low and high temperature and that sometimes low temperatures induce protection against diabetes.

Analyzing data of the English general medical practitioner for the years 2012–2014 of 4.5 million consultations of type-2 diabetic patients in England to characterize the association between ambient temperature and the disease [71], revealed that the consultations were linked to localized temperature at place of residence. Moreover, it increased during days of temperature extremes, especially during hot weather. The third study [72] monitored about half a million diabetes-associated hospitalizations in Brazil during 2000–2015. It was found that every increase of 5 °C in daily mean temperature was associated with 6% increase in hospitalization due to diabetes. Based on their data, and assuming a causal association, the authors reached an estimate that 7.3% of all diabetes-related hospitalizations during the hot season could be attributed to heat exposure. Diabetes and climate change are interconnected, both directly and indirectly [73,74]. Therefore, it is likely that with the continuous rise of global temperatures this burden will increase in particular in patients with cardiovascular complications [73].

## 7. Global Warming and Type 2 Diabetes—Potential Mechanisms

It is currently accepted that type 2 diabetes originates from concomitant-compromised metabolism which induces inflammation and impaired insulin responsiveness, leading to deviated signaling homeostasis [75,76,77]. In this respect, it has been shown that high temperatures affect insulin activity and cause an increase in diabetes incidence, apparently by inactivation of brown adipose tissue (of which the primary function is thermoregulation) [78].

As already mentioned above, HSPs are overexpressed by all cells exposed to environmental stress such as heat. In patients with diabetes these chaperones could aid improving some complications such as oxidative stress and inflammation [77], but due to the homeostasis loss HSPs levels are reduced [75,77]. In particular, both the expression of the HSP72 gene and insulin-stimulated glucose uptake are lower in diabetic patients [79]. Intriguingly, it has been shown that short exposure to heat, e.g., sauna and hot tub, reduces diabetes-related parameters including fasting glycemia, HbA1c (glycated hemoglobin), body weight, and adiposity, apparently *via* increasing HSP70 expression [80]. Taken together, stimulation of HSPs genes may become a new pharmacological intervention to improve insulin resistance [79].

Altogether we raise two potential mediators of the effect of exposure to ambient hot environment on the incidence of type 2 diabetes, brown adipose tissue metabolism and HSPs.

## 8. Conclusions

The review summarizes reports of the relationship between global warming (GW) and complex disorders, concentrating on mental disorders, primary hypertension, and type-2 diabetes, and points at potential mechanisms mediating the effect of GW on these disorders. Studies indicate that GW affects complex disorders. In general, high temperatures pose significant risk on MD. Namely, while mania is significantly increased with high temperatures, depression cases are elevated in winter. The changes involve HSPs‘ brain levels, ROS levels, and BBB breakdown. For hypertension, since the kidneys participate in regulating blood volume and blood pressure, they are the assumed target. In addition, we relate to autoimmunity, inflammation, sodium excretion, and HSP70 as risk factors. For T2D, two potential mediators are discussed—brown adipose tissue metabolism and HSPs. Generally, it seems that high temperatures also increase the incidence of diabetes cases but the relation between heat and BP is unclear. Definitive physiological mechanisms that mediate the effects of extreme weather on these complex disorders are yet unknown. Given the acceleration in climate change, they require an urgent research attention. We suggest that one such relevant direction might be the association between environmental temperature and human epigenetic modifications since there is just a minute piece of information supporting such a relationship, as recently reviewed [81].

## Data Availability

Not applicable.

## References

[1] Ebi K.L. (2022). Managing climate change risks is imperative for human health. Nat. Rev. Nephrol..

[2] Vogel M.M., Zscheischler J., Wartenburger R., Dee D., Seneviratne S.I. (2019). Concurrent 2018 Hot Extremes Across Northern Hemisphere Due to Human-Induced Climate Change. Earths Futur..

[3] Craig J. (2008). Complex diseases. Res. Appl..

[4] Bolton D. (2008). What is Mental Disorder? An Essay in Philosophy, Science, and Values.

[5] U.S. Department of Health and Human Services, Substance Abuse and Mental Health Services Administration (2021). Center for Behavioral Health Statistics and Quality Key Substance Use and Mental Health Indicators in the United States: Results from the 2020 National Survey on Drug Use and Health.

[6] Zhou B., Perel P., Mensah G.A., Ezzati M. (2021). Global epidemiology, health burden and effective interventions for elevated blood pressure and hypertension. Nat. Rev. Cardiol..

[7] Galicia-Garcia U., Benito-Vicente A., Jebari S., Larrea-Sebal A., Siddiqi H., Uribe K.B., Ostolaza H., Martin C. (2020). Pathophysiology of Type 2 Diabetes Mellitus. Int. J. Mol. Sci..

[8] Kirkman M.S., Briscoe V.J., Clark N., Florez H., Haas L.B., Halter J.B., Huang E.S., Korytkowski M.T., Munshi M.N., Odegard P.S. (2012). Diabetes in older adults: A consensus report. J. Am. Geriatr. Soc..

[9] Einarson T.R., Acs A., Ludwig C., Panton U.H. (2018). Prevalence of cardiovascular disease in type 2 diabetes: A systematic literature review of scientific evidence from across the world in 2007–2017. Cardiovasc. Diabetol..

[10] Obradovich N., Migliorini R., Paulus M.P., Rahwan I. (2018). Empirical evidence of mental health risks posed by climate change. Proc. Natl. Acad. Sci. USA.

[11] Peng Z., Wang Q., Kan H., Chen R., Wang W. (2017). Effects of ambient temperature on daily hospital admissions for mental disorders in Shanghai, China: A time-series analysis. Sci. Total Environ..

[12] Almendra R., Loureiro A., Silva G., Vasconcelos J., Santana P. (2019). Short-term impacts of air temperature on hospitalizations for mental disorders in Lisbon. Sci. Total Environ..

[13] Mullins J.T., White C. (2019). Temperature and mental health: Evidence from the spectrum of mental health outcomes. J. Health Econ..

[14] Lee H.J., Kim L., Joe S.H., Suh K.Y. (2002). Effects of season and climate on the first manic episode of bipolar affective disorder in Korea. Psychiatry Res..

[15] Chen N.T., Lin P.H., Guo Y.L. (2019). Long-term exposure to high temperature associated with the incidence of major depressive disorder. Sci. Total Environ..

[16] Ranson M. (2014). Crime, weather, and climate change. J. Environ. Econ. Manag..

[17] Krug E.G., Mercy J.A., Dahlberg L.L., Zwi A.B. (2002). The world report on violence and health. Lancet.

[18] Palinkas L.A., Wong M. (2020). Global climate change and mental health. Curr. Opin. Psychol..

[19] Hayes K., Blashki G., Wiseman J., Burke S., Reifels L. (2018). Climate change and mental health: Risks, impacts and priority actions. Int. J. Ment. Health Syst..

[20] Cianconi P., Betro S., Janiri L. (2020). The Impact of Climate Change on Mental Health: A Systematic Descriptive Review. Front. Psychiatry.

[21] Berry H.L., Bowen K., Kjellstrom T. (2010). Climate change and mental health: A causal pathways framework. Int. J. Public Health.

[22] Horowitz M. (2014). Heat acclimation, epigenetics, and cytoprotection memory. Compr. Physiol..

[23] Periard J.D., Racinais S., Sawka M.N. (2015). Adaptations and mechanisms of human heat acclimation: Applications for competitive athletes and sports. Scand J. Med. Sci. Sports.

[24] Domanico S.Z., DeNagel D.C., Dahlseid J.N., Green J.M., Pierce S.K. (1993). Cloning of the gene encoding peptide-binding protein 74 shows that it is a new member of the heat shock protein 70 family. Mol. Cell Biol..

[25] Giebel L.B., Dworniczak B.P., Bautz E.K. (1988). Developmental regulation of a constitutively expressed mouse mRNA encoding a 72-kDa heat shock-like protein. Dev. Biol..

[26] Amorim F.T., Fonseca I.T., Machado-Moreira C.A., Magalhaes Fde C. (2015). Insights into the role of heat shock protein 72 to whole-body heat acclimation in humans. Temperature.

[27] Akerfelt M., Morimoto R.I., Sistonen L. (2010). Heat shock factors: Integrators of cell stress, development and lifespan. Nat. Rev. Mol. Cell Biol..

[28] Hung J.J., Cheng T.J., Chang M.D., Chen K.D., Huang H.L., Lai Y.K. (1998). Involvement of heat shock elements and basal transcription elements in the differential induction of the 70-kDa heat shock protein and its cognate by cadmium chloride in 9L rat brain tumor cells. J. Cell Biochem..

[29] Li G.C., Mivechi N.F., Weitzel G. (1995). Heat shock proteins, thermotolerance, and their relevance to clinical hyperthermia. Int. J. Hyperth..

[30] Raichle M.E., Gusnard D.A. (2002). Appraising the brain’s energy budget. Proc. Natl. Acad. Sci. USA.

[31] Sokoloff L. (1999). Energetics of functional activation in neural tissues. Neurochem. Res..

[32] Watts M.E., Pocock R., Claudianos C. (2018). Brain Energy and Oxygen Metabolism: Emerging Role in Normal Function and Disease. Front. Mol. Neurosci..

[33] Cadenas E., Davies K.J. (2000). Mitochondrial free radical generation, oxidative stress, and aging. Free Radic Biol. Med..

[34] Katschinski D.M., Boos K., Schindler S.G., Fandrey J. (2000). Pivotal role of reactive oxygen species as intracellular mediators of hyperthermia-induced apoptosis. J. Biol. Chem..

[35] El-Orabi N.F., Rogers C.B., Edwards H.G., Schwartz D.D. (2011). Heat-induced inhibition of superoxide dismutase and accumulation of reactive oxygen species leads to HT-22 neuronal cell death. J. Therm. Biol..

[36] Freeman M.L., Spitz D.R., Meredith M.J. (1990). Does heat shock enhance oxidative stress? Studies with ferrous and ferric iron. Radiat. Res..

[37] Sies H., Jones D.P. (2020). Reactive oxygen species (ROS) as pleiotropic physiological signalling agents. Nat. Rev. Mol. Cell Biol..

[38] Vida S., Durocher M., Ouarda T.B., Gosselin P. (2012). Relationship between ambient temperature and humidity and visits to mental health emergency departments in Quebec. Psychiatr. Serv..

[39] Giles D.A., Moreno-Fernandez M.E., Stankiewicz T.E., Graspeuntner S., Cappelletti M., Wu D., Mukherjee R., Chan C.C., Lawson M.J., Klarquist J. (2017). Thermoneutral housing exacerbates nonalcoholic fatty liver disease in mice and allows for sex-independent disease modeling. Nat. Med..

[40] Hanin I. (1996). The Gulf War, stress and a leaky blood-brain barrier. Nat. Med..

[41] Patel J.P., Frey B.N. (2015). Disruption in the Blood-Brain Barrier: The Missing Link between Brain and Body Inflammation in Bipolar Disorder?. Neural Plast..

[42] McEwen B.S., Wingfield J.C. (2010). What is in a name? Integrating homeostasis, allostasis and stress. Horm. Behav..

[43] Garcia-Elias A., Mrkonjic S., Jung C., Pardo-Pastor C., Vicente R., Valverde M.A. (2014). The TRPV4 channel. Handb. Exp. Pharmacol..

[44] Shibasaki K., Sugio S., Takao K., Yamanaka A., Miyakawa T., Tominaga M., Ishizaki Y. (2015). TRPV4 activation at the physiological temperature is a critical determinant of neuronal excitability and behavior. Pflugers Arch..

[45] Lipski J., Park T.I., Li D., Lee S.C., Trevarton A.J., Chung K.K., Freestone P.S., Bai J.Z. (2006). Involvement of TRP-like channels in the acute ischemic response of hippocampal CA1 neurons in brain slices. Brain Res..

[46] Benfenati V., Amiry-Moghaddam M., Caprini M., Mylonakou M.N., Rapisarda C., Ottersen O.P., Ferroni S. (2007). Expression and functional characterization of transient receptor potential vanilloid-related channel 4 (TRPV4) in rat cortical astrocytes. Neuroscience.

[47] Konno M., Shirakawa H., Iida S., Sakimoto S., Matsutani I., Miyake T., Kageyama K., Nakagawa T., Shibasaki K., Kaneko S. (2012). Stimulation of transient receptor potential vanilloid 4 channel suppresses abnormal activation of microglia induced by lipopolysaccharide. Glia.

[48] Hatano N., Suzuki H., Itoh Y., Muraki K. (2013). TRPV4 partially participates in proliferation of human brain capillary endothelial cells. Life Sci..

[49] Jie P., Hong Z., Tian Y., Li Y., Lin L., Zhou L., Du Y., Chen L., Chen L. (2015). Activation of transient receptor potential vanilloid 4 induces apoptosis in hippocampus through downregulating PI3K/Akt and upregulating p38 MAPK signaling pathways. Cell Death Dis..

[50] Narita K., Sasamoto S., Koizumi S., Okazaki S., Nakamura H., Inoue T., Takeda S. (2015). TRPV4 regulates the integrity of the blood-cerebrospinal fluid barrier and modulates transepithelial protein transport. FASEB J..

[51] Halonen J.I., Zanobetti A., Sparrow D., Vokonas P.S., Schwartz J. (2011). Relationship between outdoor temperature and blood pressure. Occup. Environ. Med..

[52] Stotz A., Rapp K., Oksa J., Skelton D.A., Beyer N., Klenk J., Becker C., Lindemann U. (2014). Effect of a brief heat exposure on blood pressure and physical performance of older women living in the community-a pilot-study. Int. J. Environ. Res. Public Health.

[53] Xu D., Liu F., Ban J., Zhang Y., Li T. (2017). Short-term effects of ambient temperature on blood pressure: A systematic review and meta-analysis. Cardiol. Plus.

[54] Kunutsor S.K., Powles J.W. (2010). The effect of ambient temperature on blood pressure in a rural West African adult population: A cross-sectional study. Cardiovasc. J. Afr..

[55] Rodriguez-Iturbe B., Lanaspa M.A., Johnson R.J. (2019). The role of autoimmune reactivity induced by heat shock protein 70 in the pathogenesis of essential hypertension. Br. J. Pharmacol..

[56] Crandall C.G., Wilson T.E. (2015). Human cardiovascular responses to passive heat stress. Compr. Physiol..

[57] Pons H., Ferrebuz A., Quiroz Y., Romero-Vasquez F., Parra G., Johnson R.J., Rodriguez-Iturbe B. (2013). Immune reactivity to heat shock protein 70 expressed in the kidney is cause of salt-sensitive hypertension. Am. J. Physiol. Renal. Physiol..

[58] Kang Y., Han Y., Guan T., Wang X., Xue T., Chen Z., Jiang L., Zhang L., Zheng C., Wang Z. (2020). Clinical blood pressure responses to daily ambient temperature exposure in China: An analysis based on a representative nationwide population. Sci. Total Environ..

[59] Park S., Kario K., Chia Y.C., Turana Y., Chen C.H., Buranakitjaroen P., Nailes J., Hoshide S., Siddique S., Sison J. (2020). The influence of the ambient temperature on blood pressure and how it will affect the epidemiology of hypertension in Asia. J. Clin. Hypertens..

[60] Jehn M., Appel L.J., Sacks F.M., Miller E.R., DASH Collaborative Research Group (2002). The effect of ambient temperature and barometric pressure on ambulatory blood pressure variability. Am. J. Hypertens..

[61] Wang Q., Li C., Guo Y., Barnett A.G., Tong S., Phung D., Chu C., Dear K., Wang X., Huang C. (2017). Environmental ambient temperature and blood pressure in adults: A systematic review and meta-analysis. Sci. Total Environ..

[62] Peters A., Schneider A. (2021). Cardiovascular risks of climate change. Nat. Rev. Cardiol..

[63] Johnson R.J., Sanchez-Lozada L.G., Newman L.S., Lanaspa M.A., Diaz H.F., Lemery J., Rodriguez-Iturbe B., Tolan D.R., Butler-Dawson J., Sato Y. (2019). Climate Change and the Kidney. Ann. Nutr. Metab..

[64] Glaser J., Lemery J., Rajagopalan B., Diaz H.F., Garcia-Trabanino R., Taduri G., Madero M., Amarasinghe M., Abraham G., Anutrakulchai S. (2016). Climate Change and the Emergent Epidemic of CKD from Heat Stress in Rural Communities: The Case for Heat Stress Nephropathy. Clin. J. Am. Soc. Nephrol..

[65] Ephraim R.K.D., Asamoah C.A., Abaka-Yawson A., Kwadzokpui P.K., Adusei S. (2020). Climate change causes changes in biochemical markers of kidney disease. BMC Nephrol..

[66] Katori M., Majima M. (2006). A missing link between a high salt intake and blood pressure increase. J. Pharmacol. Sci..

[67] Rodriguez-Iturbe B., Pons H., Johnson R.J. (2017). Role of the Immune System in Hypertension. Physiol. Rev..

[68] Rodriguez-Iturbe B., Pons H., Quiroz Y., Johnson R.J. (2014). The immunological basis of hypertension. Am. J. Hypertens..

[69] Janeway C.A., Travers P., Walport M., Capra D.J. (2001). Immunobiology: The Immune System in Health and Disease.

[70] Seposo X.T., Dang T.N., Honda Y. (2017). How Does Ambient Air Temperature Affect Diabetes Mortality in Tropical Cities?. Int. J. Environ. Res. Public Health.

[71] Hajat S., Haines A., Sarran C., Sharma A., Bates C., Fleming L.E. (2017). The effect of ambient temperature on type-2-diabetes: Case-crossover analysis of 4+ million GP consultations across England. Environ. Health.

[72] Xu R., Zhao Q., Coelho M., Saldiva P.H.N., Zoungas S., Huxley R.R., Abramson M.J., Guo Y., Li S. (2019). Association between Heat Exposure and Hospitalization for Diabetes in Brazil during 2000-2015: A Nationwide Case-Crossover Study. Environ. Health Perspect..

[73] Zilbermint M. (2020). Diabetes and climate change. J. Community Hosp. Intern. Med. Perspect..

[74] Auliyani D., Wahyuningrum N., Supangat A.B., Basuki T.M. The bidirectional interaction between climate change and type 2 diabetes burden. Proceedings of the IOP Conference Series: Earth and Environmental Science, the 7th International Conference on Climate Change.

[75] Alemi H., Khaloo P., Rabizadeh S., Mansournia M.A., Mirmiranpour H., Salehi S.S., Esteghamati A., Nakhjavani M. (2019). Association of extracellular heat shock protein 70 and insulin resistance in type 2 diabetes; independent of obesity and C-reactive protein. Cell Stress Chaperones.

[76] Hooper P.L., Hooper P.L. (2009). Inflammation, heat shock proteins, and type 2 diabetes. Cell Stress Chaperones.

[77] Zilaee M., Shirali S. (2016). Heat Shock Proteins and Diabetes. Can. J. Diabetes.

[78] Blauw L.L., Aziz N.A., Tannemaat M.R., Blauw C.A., de Craen A.J., Pijl H., Rensen P.C.N. (2017). Diabetes incidence and glucose intolerance prevalence increase with higher outdoor temperature. BMJ Open Diabetes Res. Care.

[79] Koranyi L., Hegedus E., Peterfal E., Kurucz I. (2004). The role of hsp60 and hsp70 kDa heat shock protein families in different types of diabetes mellitus. Orvosi Hetil..

[80] Krause M., Ludwig M.S., Heck T.G., Takahashi H.K. (2015). Heat shock proteins and heat therapy for type 2 diabetes: Pros and cons. Curr. Opin. Clin. Nutr. Metab. Care.

[81] Xu R., Li S., Guo S., Zhao Q., Abramson M.J., Li S., Guo Y. (2020). Environmental temperature and human epigenetic modifications: A systematic review. Environ. Pollut..

